# Malignant tumours of the salivary glands

**DOI:** 10.1054/bjoc.2001.1762

**Published:** 2001-05

**Authors:** R J Bensadoun, M P Blanc-Vincent, P Chauvel, O Dassonville, G Gory-Delabaere, F Demard

**Affiliations:** Centre Antoine Lacassagne, Nice; Cancer Research Campaign, Paris; Institut Bergonié, Bordeaux; Centre Val d'Aurelle Paul-Lamarque, Montpellier, France


					Malignant tumours of the salivary glands are relatively rare, with
an estimated incidence of less than 1 per 100 000. They represent a
little less than 5% of head and neck tumours. These are generally
slow-growing tumours of varied histology, long doubling times
and late locoregional and distant recurrences. Prolonged survival
is possible, even with metastatic disease.
These recommendations do not consider salivary gland
lymphomas. They were validated in August 1999 and an update is
planned for the year 2000.
PROGNOSTIC FACTORS
The most important prognostic factors with respect to local control
and survival are tumour size and clinical stage. Other independent
factors are the histology, (low grade vs high grade) and the treat-
ment (the quality of the surgical excision). Knowledge of these
factors is part of initial staging, and allows the subsequent thera-
peutic strategy to be matched to the clinical situation.
DIAGNOSIS AND STAGING
Standard staging of malignant tumours of the salivary glands is
based on clinical examination, imaging and histopathological
examination.
From clinical examination, the size of the lesion, locoregional
extension, and any signs suggestive of malignancy (e.g. facial
paralysis, trismus, cutaneous infiltration) can be determined.
Endoscopic examination (often under general anaesthesia) is
required to obtain biopsy samples and to complete the staging of
tumours of the minor salivary glands (notably those in the pharynx
and larynx).
Clinical assessment of locoregional extension is based on exam-
ination of the neck. A total body examination is required to look
for distant metastases. An assessment of performance status, nutri-
tional status and of major organ function, using clinical and labor-
atory parameters, determines whether or not the patient has
operable disease (standard).
Standard imaging consists of a cervico-facial CT scan or high-
resolution ultrasound (level of evidence B). High-resolution ultra-
sonography must only be used by those teams trained in this
method. For tumours under the maxilla, an orthopantogram is neces-
sary to complete staging (standard). MRI imaging and sialograms
are options. Further imaging is done according to symptoms and
signs suggestive of malignant involvement and extraglandular
spread.
Histopathological examination is necessary to confirm a diag-
nosis of malignancy. An excision biopsy with frozen section is
required for the major salivary glands (standard) and a simple
biopsy for the minor salivary glands (standard). A preoperative
diagnosis can be made by cytological analysis following fine-
needle aspiration (option).
Staging
The TNM AJC/UICC classification is the most practical and the
best adapted to treatment decision-making (standard). The histo-
logical classification differentiating the tumours according to
grade (low vs high grade), is now universally recognized (stan-
dard).
TREATMENT
The basic treatment of salivary gland tumours is complete surgical
excision (standard), with or without postoperative irradiation,
according to the clinical stage and the histological grade. The
combination of surgery and radiotherapy is the treatment of choice
for high grade disease.
Routine postoperative radiotherapy is indicated for stage II, III
and IV high grade tumours and for low grade stage III and
IV tumours (standard). It is also indicated in all cases in
which surgery has been macro- or microscopically incomplete
(standard).
Neutron therapy alone, when possible, is the treatment of choice
for inoperable tumours, whatever the stage and grade. To a lesser
degree, neutron therapy provides an alternative treatment for
locally advanced disease (stages III and IV) where surgery is likely
to be difficult or to result in significant functional sequelae
(option). This requires further evaluation. Postoperative neutron
therapy is not indicated except in the case of large-volume residual
disease (option, level of evidence C).
The place of chemotherapy remains unclear. It should only be
given within a multicentre therapeutic trial. Treatment is according
to clinical stage and histological grade.
Stage I, low grade / high grade: T1a T2a N0 M0
Complete surgical resection is the standard treatment for stage I
tumours of the salivary glands (Figures 1 and 2). For tumours of
the major salivary glands the gland must be completely excised
(standard). For tumours of the minor salivary glands, a wide
radical resection must be undertaken (standard). In all cases, the
excision should be complete and in the case of encapsulated
tumours, outside the capsule.
If the resection is macroscopically and microscopically
complete, there is no indication for adjuvant radiotherapy, even for
high grade tumours (standard).
If the excision is incomplete, or if histological examination has
shown tumour at the excision margins, postoperative radiotherapy
is indicated (standard). Postoperative irradiation should be with
photons (electrons) with standard fractionation (level of evidence
A). Irradiation with neutrons can be given in cases of large-volume
residual disease (option, level of evidence C).
Malignant tumours of the salivary glands
RJ Bensadoun1
, MP Blanc-Vincent2,3
, P Chauvel1
, O Dassonville1
, G Gory-Delabaere2,4
and F Demard1
1
Centre Antoine Lacassagne, Nice; 2
FNCLCC, Paris; 3
Institut Bergonie, Bordeaux; 4
Centre Val d'Aurelle Paul-Lamarque, Montpellier, France
42
British Journal of Cancer (2001) 84(Supplement 2), 42-48
(c) 2001 FNCLCC
doi: 10.1054/ bjoc.2001.1762, available online at http://www.idealibrary.com on
Malignant tumours of the salivary glands 43
British Journal of Cancer (2001) 84 (Supplement 2), 42-48
(c) 2001 FNCLCC
Routine ipsilateral nodal clearance is standard for T2 high grade
tumours (standard, level of evidence B) and is an option for T1a
tumours (option). If nodal involvement is detected at the time of
surgery, ipsilateral neck dissection followed by routine postopera-
tive radiotherapy is recommended (level of evidence B).
Stage II, T1b T2b T3a N0 M0
Low grade tumours
Surgery alone, of variable extent, is the standard treatment (Figure 3).
If a complete resection is likely to result in a significant functional
or cosmetic deficit, neutron therapy alone can be undertaken
(option, level of evidence C). Postoperative radiotherapy must be
undertaken if the surgical excision margins are involved (stan-
dard). In the case of positive nodes, extended surgical excision of
the tumour and cervical nodes is followed by postoperative irradi-
ation irrespective of whether the resection is macroscopically or
microscopically complete (level of evidence B) or incomplete
(level of evidence A).
High grade tumours
Surgery alone (complete excision, with or without subsequent
extraglandular extension and ipsilateral neck dissection) is indi-
cated for lesions limited to the gland (standard) (Figure 4). In the
case of extension of the tumour to involve the facial nerve or
crucial structures, the therapeutic options are extensive disfiguring
surgery or neutron therapy alone. External irradiation (with
neutrons) as first-line therapy can be considered for lesions judged
to be incompletely resectable, or where extensive disfiguring
surgery will be necessary with the risk of significant functional
impairment (notably disease extending to the facial nerve, infra-
temporal fossa or the mandible) (option, level of evidence B).
Routine postoperative external irradiation to tumour and nodes
is standard when the excision has been incomplete (macro- and
microscopically) or in the case of positive nodes (standard). It is
recommended in cases of complete excision (option, level of
evidence B). The role of chemotherapy, either as neoadjuvant or
adjuvant treatment or with the aim of radiosensitization, must be
evaluated in prospective therapeutic trials (it has no role as routine
treatment).
Stage III, T3b T4a N0 M0 all T (except T4b) N1 M0
Low grade tumours
Low grade stage III tumours are treated in the same way as high
grade stage II tumours (Figure 4). If surgery has been complete,
postoperative irradiation to the tumour and nodes remains the stan-
dard treatment. Neutron therapy alone is preferable in certain cases
where surgery would be very extensive and disfiguring. The stan-
dard treatment of N1 lesions is primary tumour and nodal surgery
with uni- or bilateral neck dissection (bilateral in the case of
midline tumours), followed by postoperative irradiation (stan-
dard). The extent of nodal irradiation will depend on the histo-
logical findings following neck dissection.
High grade tumours
For stage III high grade tumours, two types of treatment can be
considered: complete excision of the primary tumour and nodes,
followed by radiotherapy, or neutron therapy alone to both tumour
and nodes (option) (Figure 5). The place of chemotherapy must be
evaluated within prospective trials (it has no role as routine treat-
ment).
Stage IV disease
T4b low and high grade, all N, M0
There is no standard therapeutic approach for the management of
patients presenting with stage 4b low or high grade node-positive
stage IV disease (Figure 6).
Stage 1 (T1a, N0 M0, and T2a N0 M0)
Low grade tumours
resection macroscopically and
microscopically complete?
Yes no
Standards
* major gland tumours: complete excision of the gland
* minor gland tumours: extended excision
Recommendation
no routine neck dissection
Follow-up
Follow-up
Standard
Postoperative radiotherapy (RT) (photons/electrons)
Option
neutron therapy alone for macroscopic residual disease
Recommendations:
* standard fractionation
* radiotherapy to tumour bed and ipsilateral cervical
nodal areas
Figure 1 Treatment of stage I carcinoma of the salivary glands (low grade)
Stage 1 (T1a,N0 M0, and T2a N0 M0)
High grade tumours
Standards
* major gland tumours: complete surgical excision
* minor gland tumours: surgical excision
* T2a tumours: ipsilateral neck dissection
Option
T1a tumours: ipsilateral neck dissection
Recommendation
nerves should not be conserved at the expense of tumour clearance
Follow-up
Follow-up
complete macro- and microscopic excision
and
no evidence of microscopic invasion of
perineural tissue and/ or soft tissue (pT1b)
and/or N+(pN1)?
Standard
postoperative radiotherapy to tumour and
nodes (photons/electrons)
Option
postoperative neutron therapy alone in the
case of large volume residual disease
yes no
Figure 2 Treatment of stage I disease (high grade)
44 RJ Bensadoun et al
British Journal of Cancer (2001) 84 (Supplement 2), 42-48 (c) 2001 FNCLCC
The therapeutic options are neutron therapy alone to tumour and
nodes or extensive disfiguring surgery followed by radiotherapy
(level of evidence B).
Surgery should be radical and carried out by cancer specialists.
The role of chemotherapy remains to be evaluated within prospec-
tive therapeutic trials.
For T4b N0 M0 disease, neutron therapy alone is the technique
of choice (level of evidence B). The role of chemotherapy must be
evaluated within prospective therapeutic trials (no role in routine
use).
All T, N2 or N3 M0 disease
If the primary lesion is easily resectable (T1-T2), the standard
treatment is surgical excision of tumour and nodes. In all other
cases, neutron therapy alone, when possible, is preferable to exten-
sive disfiguring surgery (option) (Figure 6).
All T, all N, M1 disease (distant metastases)
In metastatic disease, the recommended therapeutic approach is
palliative treatment (standard) and the evaluation of chemotherapy
and/or surgery and/or radiotherapy within multicentre trials (option)
(Figure 7). Elective surgical excision is the standard treatment for
isolated pulmonary metastases.
Inoperable disease
In patients unfit for surgery, or with unresectable disease, the
recommended treatment is neutron therapy alone where possible
(option, level of evidence B) (Figure 8). Standard radiotherapy
with photons, including those protocols using hyperfractionation
or accelerated irradiation (which is still under evaluation in this
condition) gives inferior results (level of evidence B).
Stage II:T1b,T2b,T3a,N0 M0
Low grade tumours
Extension to vital structures?
Standard
complete excision
Option
T3a tumours: ipsilateral
neck dissection
Standard
there is no standard
Option
radical surgery
Option
neutron therapy alone
Resection macro- and
microscopically complete
and
no involved nodes?
no
no
yes
yes
Standard
postoperative radiotherapy (photons/electrons) in
standard doses and fractions to tumour and
nodes
Option
postoperative neutron therapy for large volume
residual disease
Follow-up
Follow-up
Figure 3 Treatment of stage II disease (low grade)
Incomplete surgery followed by radiotherapy with photons, is
not recommended. The place of chemotherapy, especially in high
grade tumours, must be evaluated within prospective trials.
Loco-regional recurrence
There is no standard therapeutic approach. This will depend
largely on the type of recurrence and above all on the treatment
that has previously been given to the primary tumour. In the
case of relapse after surgery, further surgery followed by postoper-
ative radiotherapy, or neutron therapy alone can be considered
(options). For recurrent disease after radiotherapy alone or after
surgery followed by radiotherapy, the treatment options are: repeat
surgery if possible, neutron therapy at a dose limited by the
previous irradiation, or chemotherapy within a prospective trial
(especially for high grade disease) (Figure 9).
For nodal relapses, uni- or bilateral nodal dissection ( post-
operative irradiation), can be considered (options). If the recurrence
is inoperable, neutron therapy alone, irradiation with photons
combined with localized hyperthermia, or chemotherapy within a
prospective trial (especially for high grade disease) are the treatment
options.
FOLLOW-UP
Monthly surveillance is recommended during the first 6 months
following treatment (3 months in the case of low grade tumours
and stage I and II disease). Thereafter, follow-up can be 4-monthly,
then 6-monthly for 3-4 years, then annually. The assessment
should include a chest X-ray (AP and lateral) every 6 months
initially, then every year.
INTERNAL REVIEWERS
C Alzieu (Institut Paoli Calmettes, Marseille), P Barrellier (Centre
Francois Baclesse, Caen), G Dolivet (Centre Alexis Vautrin,
Malignant tumours of the salivary glands 45
British Journal of Cancer (2001) 84 (Supplement 2), 42-48
(c) 2001 FNCLCC
Stage II, high grade tumours
or Stage III (T3b,T4a,N0 M0; All T except T4b N1 M0)
Low grade tumours
Lesion limited to the gland
with no extension to vital structures?
Standard
surgery: complete excision
with ipsilateral neck dissection
Standard
there is no standard
Option
radical surgery
Option
Neutron therapy alone
Complete macro-
and
microscopic resection with no
involved nodes?
Standard
postoperative radiotherapy to tumour and nodes
Options
* radiotherapy with photons/electrons
* neutron therapy for macroscopic residual disease
* chemotherapy within a trial
Recommendation
for photon/electron radiotherapy:
standard does and fractionation, 65 Gy to tumour
bed, dose to ipsilateral cervical nodes according to N
status
Standard
there is no standard
Options
* postoperative radiotherapy
* no treatment
Follow-up
no yes
yes no
Figure 4 Treatment of stage II disease (high grade) or stage III (low grade)
46 RJ Bensadoun et al
British Journal of Cancer (2001) 84 (Supplement 2), 42-48 (c) 2001 FNCLCC
Vandoeuvre-les-Nancy), M Fabbro (Centre Val d'Aurelle,
Montpellier), L. Geoffrois (Centre Alexis Vautrin, Nancy),
B Gignoux (Centre Regional Leon Berard, Lyon), JM Hannoun-
Levi (Institut Paoli Calmettes, Marseille), JC Horiot (Centre
Georges-Francois Leclerc, Dijon), P. Marandas (Institut Gustave
Roussy, Villejuif), F Pech-Gourg (Institut Paoli Calmettes,
Marseille), JL Renaud-Salis (Institut Bergonie, Bordeaux),
J Rodriguez (Institut Curie, Paris), G Schwaab (Institut Gustave
Roussy, Villejuif), T Tursz (Institut Gustave Roussy, Villejuif) and
P Wibault (Institut Gustave Roussy, Villejuif).
EXTERNAL REVIEWERS
N Barbet (Macon), E Barthelme (Hopital Bon Secours, Metz),
I Bartholomot (Saint-Nazaire), P Bergerot (Saint-Nazaire),
P Bertoin (Lyon) M Bethouart (Cabinet d'Anatomocytopathologie,
Roubaix), M Boukhefila (Wattrelos), N Breteau (CHR Hopital de
la Source, Orleans), P Breton (Centre Hospitalier Lyon Sud,
Pierre-Benite), JF Chassagne (CHU Hopital Central, Nancy),
R Combelles (Hopital Purpan, Toulouse), R Coquard (Clinique
Saint-Jean, Lyon), P Desprez (Centre Saint-Yves, Vannes),
M Drey (Reims), F Economides (Dunkerque), D Fric (Clinique du
Mail, Grenoble), B Guerrier (Hopital Saint-Charles, Montpellier),
D Heymans (Centre Saint-Michel, La Rochelle), M Housset
(Hopital Tenon, Paris), P Juin (CHU Hopital la Timone,
Marseille), G Le Clech (Hopital de Pontchaillou, Rennes),
P Martin (Clinique Saint-Jean, Lyon), KT Nguyen (Roubaix),
C Pasteris (Clinique de Savoie, Annemasse), JL Reynoard
(Clinique Victor Hugo, Angouleme), N Rouverand (Clinique de la
Louviere, Lille), P Seguin (CHU Hopital de Bellevue, Saint-
Etienne), F Siberchicot (Hopital Pellegrin Tripode, Bordeaux) and
J Trotoux (Hopital Boucicaut, Paris).
Stage III (T3b,T4a N0 M0
all T except T4b N1 M0)
high grade tumours
Standard
there is no standard
Option
complete surgery plus ipsilateral neck dissection
Option
neutron therapy alone
Standard
routine postoperative radiotherapy to tumour and nodes
Options
* postoperative neutron therapy for bulky residual disease
* chemotherapy (adjuvant, neoadjuvant ) within a clinical trial
Recommendation
standard dose and fractionation, tumour dose 65 Gy, dose to nodes according to N
status
Follow-up
Figure 5 Treatment of stage III disease (high grade)
Malignant tumours of the salivary glands 47
British Journal of Cancer (2001) 84 (Supplement 2), 42-48
(c) 2001 FNCLCC
Non-resectable tumours
patients not fit for surgery
all TNM
Standard
there is no standard
Options
* neutron therapy alone
* chemotherapy for high grade disease within a clinical trial
Follow-up
Figure 8 Treatment of inoperable disease
Stage IV
Non-metastatic disease
All T N2 or N3 T4b, all grades
Resectable tumour
(T1-T2)?
no
no
yes
yes
Standard
there is no standard
Standard
surgery to tumour and nodes
Option
complete excision
and neck dissection
Option
neutron therapy alone
Standard
routine postoperative radiotherapy
to tumour and nodes
Standard
there is no standard
Options
* radical surgery followed by
radiotherapy to tumour and nodes
* neutron therapy alone to tumour
and nodes
* chemotherapy within a clinical trial
Follow-up
Figure 6 Treatment of stage IV non-metastatic disease
Stage IV
metastatic disease
Stage IV
metastatic disease
Resectable pulmonary metastases?
Standard
palliative treatment
Options
* radiotherapy for palliation or tumour reduction
* cytoreduction surgery
* chemotherapy (high grade tumours) within a
clinical trial
Standard
surgery
Follow-up
no yes
Figure 7 Treatment of stage IV metastatic disease
48 RJ Bensadoun et al
British Journal of Cancer (2001) 84 (Supplement 2), 42-48 (c) 2001 FNCLCC
Salivary gland tumours
Locoregional recurrence
Recurrent disease operable?
no yes
yes no
Recurrence after
surgery alone?
Standard
there is no standard therapy
Options
* neutron therapy alone
* photon radiotherapy combined
with localized hyperthemia
* chemotherapy
Standard
there is no standard therapy
Options
* further surgery and therapy
radiotherapy
* uni or bilateral neck dissection in
cases of nodal relapse
* neutron therapy alone
Standard
there is no standard therapy
Options
* further surgery
* uni or bilateral neck dissection in
case of nodal relapse
* neutron therapy at a dose limited
by prior irradiation
* chemotherapy within a clinical trial
Figure 9 Treatment of locoregional recurrence

				

